# Association between Vitamin D Deficiency and Levels of Renin and Angiotensin in Essential Hypertension

**DOI:** 10.1155/2022/8975396

**Published:** 2022-06-10

**Authors:** Lu Han, Xin-Juan Xu, Jun-Shi Zhang, Hai-Ming Liu

**Affiliations:** ^1^Department of Cardiology, The Armed Police Corps Hospital, Xinjiang, Urumqi 830000, China; ^2^Department of Hypertension, The First Affiliated Hospital of Xinjiang Medical University, Urumqi 830054, China

## Abstract

**Objective:**

The present study aims to investigate the relationship between vitamin D deficiency and renin-angiotensin-aldosterone levels in patients with essential hypertension.

**Methods:**

The present study observed two groups of patients from Urumqi, Xinjiang, China, from April 2017 to March 2018. There were two subject groups: the hypertension group (80 patients with essential hypertension selected by random cluster sampling) and the control group (76 healthy adults). The 25-hydroxyvitamin D (25(OH)D or vitamin D) levels were measured through electrolytes; fasting blood glucose, blood lipids, and other biochemical indicators were detected using immune chemiluminescence; and plasma renin activity and angiotensin II concentrations were detected with radio-immunity.

**Results:**

Comparison between the hypertension group and control group showed statistically significant differences in the systolic pressure and levels of 25(OH)D, renin, and triglycerides (*P* < 0.05). The correlation analysis showed that 25(OH)D was negatively correlated with renin (*r* = –0.185; *P*=0.021) and positively correlated with systolic pressure (*r* = –0.105; *P*=0.035). There were no statistically significant differences in diastolic pressure, fasting blood glucose, total cholesterol, low-density lipoprotein cholesterol, high-density lipoprotein cholesterol, and triglycerides between the two groups.

**Conclusions:**

The results of the present study show that vitamin D deficiency is common in Urumqi, Xinjiang, China and vitamin D levels are negatively correlated with renin levels. Vitamin D plays an important role in regulating blood pressure by affecting renin levels through the renin-angiotensin-aldosterone system.

## 1. Introduction

Hypertension is a chronic cardiovascular disease that can be caused by many factors. Long-term hypertension can damage the functions of the heart, brain, kidneys, and other target organs; it is the “number one killer,” endangering human health and life [1]. The pathogenesis of essential hypertension can result from the interaction of multiple factors, such as heredity, environment, and lifestyle. In-depth studies incorporating molecular biology technology and epidemiological theory have shown that hypertension can be related to many metabolic factors of the body; the relationship between 25-hydroxyvitamin D (25 [OH] D or vitamin D) deficiency and hypertension is especially important [2, 3]. Other studies have also shown that vitamin D and the renin-angiotensin-aldosterone system (RAAS) are closely related [4].

In Urumqi, the climate is characterized by long cold winters and severe air pollution that reduces the residents' outdoor activities; low exposure to sunlight leads to a prevalence of vitamin D deficiency [5]. The present study will investigate the correlation between vitamin D, the RAAS, and the pathogenesis of hypertension through the comparison of two groups: the hypertension group (patients with essential hypertension) and the control group (healthy subjects). The study will also discuss how vitamin D can control blood pressure by regulating the activity of RAAS, suggesting a new direction and basis for the prevention and treatment of hypertension.

### 1.1. Study Populations

The present paper is a case-control study. Via random sampling, 80 patients with essential hypertension were chosen from the Department of Hypertension of the First Affiliated Hospital of Xinjiang Medical University and assigned to the essential hypertension group and 76 healthy adults were selected from the physical examination center and assigned to the control group.

Exclusion criteria for patients with hypertension include the following: (1) patients who had taken antihypertensive drugs; (2) patients who were using contraceptives; (3) patients with secondary hypertension, diabetes, thyroid disease, kidney disease, or sleep apnea syndrome.

The present study was conducted with approval from the Ethics Committee of the First Affiliated Hospital of Xinjiang Medical University and in accordance with the Declaration of Helsinki. Written informed consent was obtained from all participants.

### 1.2. Blood Pressure Measurements

The patient's blood pressure was measured by a physician using a Mercury sphygmomanometer with a cuff of the appropriate size. The subject remained seated for 10 minutes, with the right arm comfortably placed at the level of the heart; the systolic pressure was recorded with the first phase of the Korotkoff sound, and the diastolic pressure was recorded with the fifth phase of the Korotkoff sound. Each patient was measured three times, with an interval of 2 minutes between measurements, and the values were averaged.

### 1.3. Laboratory Tests

Blood samples were obtained after overnight fasting, and the serum was stored in aliquots at –80°C until analysis. Total serum 25(OH)D was measured using a Roche Modular Analytics E170 and a commercial kit. The Roche 25-HydroxyVitamin D_3_ Kit (Electrochemiluminescence) was obtained from Roche Diagnostics GmbH, Germany (China Food and Drug Administration No. 2400865). Biochemical parameters, such as blood glucose, kidney function, and lipid profiles (total cholesterol, high-density lipoprotein (HDL) cholesterol, low-density lipoprotein (LDL) cholesterol, and triglycerides), were measured using an auto-analyzer (ADVIA1650, Siemens, NY, US) and commercially available kits.

Blood samples for plasma renin activity (PRA) and angiotensin II were first collected between 8:30 and 10:30 AM after the patients had rested for a minimum of eight hours. Patients were instructed to reduce their salt intake three days before the PRA measurement. The PRA and angiotensin II samples were collected in ice-cold vacutainer tubes containing ethylenediaminetetraacetic acid. The PRA was measured using radioimmunoassay (125I-labeled PRA, Beijing North Institute of Biological Technology, China), and plasma angiotensin II was measured using radioimmunoassay (125I-labeled angiotensin II, Beijing North Institute of Biological Technology, China).

### 1.4. Statistical Analysis

SPSS 18.0 software was used for database management and statistical analysis. Data were given as mean ± SD for continuous variables. The counting data were calculated using a chi-square test, and the measurement data were assessed for normal distribution and homogeneity of variance. Two independent sample *t*-tests were used for data conforming to a normal distribution and homogeneity of variance, and Pearson correlation was used to analyze the associations between variables. A *P* value of <0.05 was considered statistically significant.

## 2. Results

Among the patients in the hypertension group (*n* = 80), 46 were males and 34 were females and 59 were of Han nationality and 21 were of Uygur nationality. The patients had an average age of 40.04 ± 6.83 years. Among the subjects in the control group (*n* = 76), 34 were males and 42 were females and 49 were of Han nationality and 27 were of Uygur nationality. The subjects had an average age of 39.82 ± 10.07 years.

At present, there is no consensus on the standard of serum 25(OH)D; however, most studies classify its concentration levels. Serum 25(OH)D ≥ 30 (ng/ml)/75 (mol/L) was sufficient for vitamin D; 25(OH)D between 20∼30 (ng/mL) and 50∼75 (mol/L) was considered vitamin D not enough; 25(OH)D between 10∼20 (ng/mL) and 25∼50 (mol/L) was considered vitamin D deficiency; and 25(OH)*D* < 10 (ng/mL)/25 (mol/L) was considered severe vitamin D deficiency. There were 78 cases (97.5%) with vitamin D deficiency in the essential hypertension group and 67 cases (88%) with vitamin D deficiency in the control group.

There were statistically significant differences between the levels of serum 25(OH)D, systolic pressure, renin, and triglycerides between the two groups (*P* < 0.05) (Table 1).

**Figure 1:**
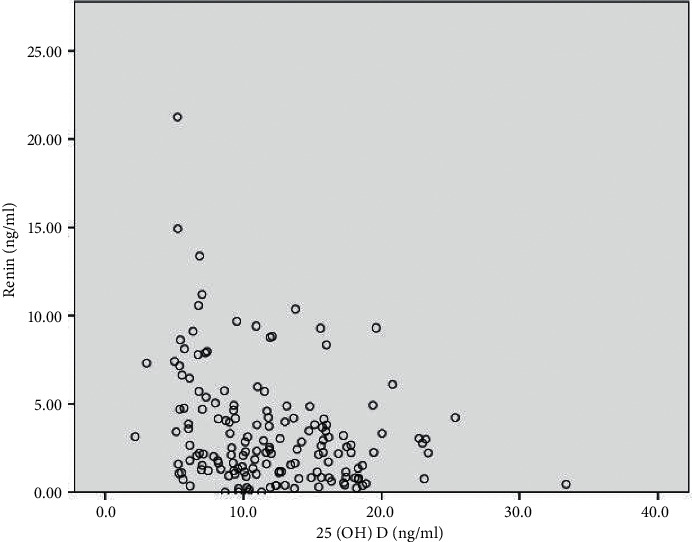
Correlation of serum 25(OH)D with renin (*r* = −0.185; *P*=0.021).

**Figure 2:**
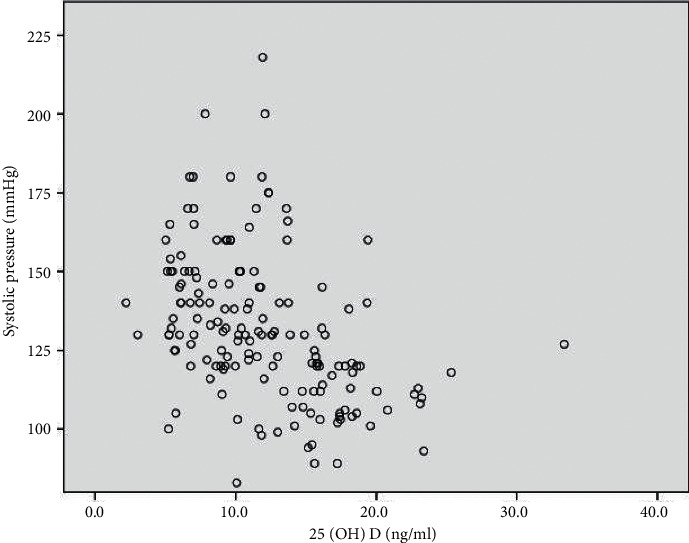
Correlation of serum 25(OH)D with systolic pressure (*r* = −0.105; *P*=0.035).

Pearson correlation was used to analyze the associations between blood pressure and serum 25(OH)D levels, RAAS components, and biochemical parameters in the hypertension group. In the correlation analysis, serum 25(OH)D levels were negatively correlated with renin (*r* = −0.185; *P*=0.021) (Figure 1) and systolic blood pressure (*r* = –0.105; *P*=0.035) (Figure 2). No correlation was found between serum 25(OH)D concentrations and diastolic blood pressure, blood glucose, total cholesterol, LDL cholesterol, HDL cholesterol, and triglyceride levels (Table 2).

## 3. Discussion

The present study showed that vitamin D deficiency is also widespread in the population of Xinjiang; this conclusion is consistent with the existing literature [5,6]. This may be related to the geographical location, climate, or diet. The latitude of Urumqi is N32°22′, and the region has long cold winters and low exposure to sunlight or type B ultraviolet light. This is consistent with the low levels of 25(OH)D found in both the hypertension group and the control group.

In this study, although there was no statistically significant difference between 25(OH)D and BMI, the BMI level of the hypertension group was higher than that of the control group, while the 25(OH)D level was lower than that of the control group, suggesting that there may be a certain relationship between 25(OH)D and BMI. A study of more than 12,000 adults [7] showed an inverse relationship between high BMI and vitamin D supplementation. Another study showed that the serum 25(OH)D level of obese people was inversely proportional to body weight, body mass index (BMI), and fat quantity [8], indicating that vitamin D supplementation was related to body weight. Vitamin D/Vitamin D receptor (VDR) system is involved in the regulation of lipid metabolism of adipocytes; leptin interacts with VDR to produce autocrine-paracrine lipolytic effect on adipocytes and controls lipid metabolism by inhibiting adipogenesis and stimulating lipid decomposition [9]. Vitamin D needs to be converted to 25(OH)D in the liver, and the synthesis of 25(OH)D is also affected once the fat in the liver is degenerated. It can be seen from this study that there is a negative correlation between BMI level and vitamin D level, indicating that obesity will lead to a decrease in vitamin D level and vitamin D level in the body will change with the change in BMI. Obesity, BMI, and hypertension are closely related, which indicates that the association and regulatory mechanisms between vitamin D and hypertension are complex and a variety of factors are involved, which directly and indirectly affect blood pressure and even the cardiovascular system.

The RAAS system, which circulates hormones synthesized and secreted by many of the body's organs, plays an important role in regulating blood pressure, maintaining the balance of water and electrolytes in the body, and preserving a stable environment [10]. Although many studies on vitamin D and hypertension have shown that low serum 25(OH)D levels are a risk factor for hypertension [2, 11, 12], the relationship between vitamin D and blood pressure is unclear.

The answer to the question regarding the correlation between vitamin D levels and blood pressure may be related to RAAS [13]. Vitamin D plays an important role in inhibiting renin secretion and may regulate RAAS activity in humans. Vitamin D could reduce the activity of the renin gene promoter [14] as well as inhibit renin secretion and the activation of RAAS throughout the body, thus reducing the angiotensin II level. This shows that vitamin D suppresses the RAAS and decreases blood pressure by inhibiting renin activity. Animal experiments have shown that circulating vitamin D can restrain renin expression in peritubular cells as well as the proliferation of vascular smooth muscle cells [15].

Vitamin D also strongly inhibits renin synthesis [16] and can exert downstream effects by binding to the vitamin D receptor (VDR). After binding to the VDR, vitamin D can connect with the cyclic adenosine monophosphate response element-binding protein to prevent renin expression [5]. The results obtained by studying cell cultures suggest that 25-(OH)_2_D_3_ can inhibit the transcription of renin mRNA through the VDR and directly inhibit the proliferation of vascular smooth muscle cells. If the 25(OH)D levels are low, the RAAS system can become activated. The overactivation of RAAS is a primary mechanism for the pathogenesis leading to hypertension. [17].

Vitamin D deficiency and RAAS activation can cause water and sodium retention, increase circulation capacity, and increase blood pressure [18]. In addition, RAAS activation leads to increased sympathetic nerve activity, peripheral vasoconstriction, and vascular endothelial dysfunction and decreased vascular elasticity and compliance, further aggravating hypertension and arteriosclerosis.

At present, one of the widely recognized mechanisms of vitamin D for lowering blood pressure is through the reduction of blood pressure by affecting the RAAS, causing the two to interact.

The present study showed no significant correlation between serum 25(OH)D concentration and the levels of fasting blood glucose, triglyceride, total cholesterol, and LDL; however, the concentration of 25(OH)D was negatively correlated with renin level, which also plays an important role in blood pressure regulation; these results are consistent with the results of previous clinical studies.

A recent cross-sectional study found a negative association between vitamin D levels and PRA in individuals with hypertension on both low-salt and high-salt diets [19]. However, no prospective human clinical trial has confirmed this association. The regulation of vitamin D on human renin expression is complex, and its mechanism is not completely understood.

The parathyroid hormone (PTH) is a peptide secreted by the main cells of the parathyroid gland that can increase the blood calcium (Ca2+) level and decrease the blood phosphorus level. Studies have shown that there is a correlation between RAAS and PTH [20]. Cross-sectional data from the United States Nutrition Survey suggest that the association between vitamin D and blood pressure is mediated by PTH [21]. Dietary vitamin D supplementation for patients with hypertension/inflammatory disease may be used to achieve PTH inhibition [22]; hence, the blood-pressure-lowering effect of vitamin D may also be partially regulated by PTH. Vitamin D supplementation may help control blood pressure by inhibiting PTH.

Hypertension is a central, peripheral, genetic, environmental, endocrine, and neurological disease. Hemodynamic factors affect the result in many aspects, and vitamin D deficiency can activate RAAS; it also affects the blood pressure of patients with hypertension. The relationship between vitamin D and blood pressure is regulated by PTH, and vitamin D deficiency may be a factor of hypertension onset; thus, it is necessary to study and understand this relationship and the regulatory mechanism between the two. Vitamin D supplementation may have a significant impact on preventing the occurrence, development, and prognosis of hypertension. It may also improve the health level of the population by intervening in high-risk hypertension groups and acting as an auxiliary means for clinical treatment of hypertension.

The current study has several limitations: (1) After excluding subjects who used drugs to interfere with RAAS, only a low number of selected subjects were studied, resulting in a small sample size. A larger study size is necessary. (2) As the survey was conducted in only one hospital, the samples were not randomly selected and may not represent the vitamin D status of all patients with hypertension in Xinjiang. (3) The present paper is a cross-sectional study and thus cannot determine the causal relationship between serum 25(OH)D, RASS, and PTH; it can only indicate that there may be mutual influence and action among these factors. (4) This paper focuses on the study of serum 25(OH), renin, and angiotensin and does not include aldosterone, PTH, and other factors related to vitamin D.

## 4. Conclusions

The present observational study suggests that vitamin D deficiency is highly prevalent in individuals with hypertension residing in Xinjiang, China. The present study provides evidence for the association between vitamin D deficiency and hypertension. A large and rigorously designed randomized clinical trial is needed to investigate whether vitamin D supplements can treat and prevent hypertension. The authors of the present study think that it may provide some basis for the prevention and treatment of hypertension.

## Figures and Tables

**Table 1:** Comparison of clinical data between the hypertension group and control group (means ± SD).

	Hypertension group (*n* = 80)	Control group (*n* = 76)	Test value	*P* value
BMI (kg/m^2^)	26.49 + 3.42	24.84 + 4.17	-3.163	0.056
Systolic pressure (mmHg)	149.5 + 22.75	116.63 + 14.91	10.619	<0.001^*∗*^
Diastolic pressure (mmHg)	98.20 + 14.76	73.63 + 11.98	11.397	0.060
25(OH)D (ng/ml)	7.96 + 2.61	9.82 + 4.70	-3.076	0.002^*∗*^
Renin (ng/ml)	5.75 + 3.17	2.57 + 2.39	7.028	<0.001^*∗*^
Ang II (ng/ml)	76.12 + 31.08	63.91 + 48.97	1.881	0.062
Creatinine (umol/ L)	66.01 + 18.71	68.19 + 34.77	-0.443	0.659
Blood glucose (mmol/L)	5.09 + 0.99	4.82 + 0.82	1.739	0.084
Triglyceride (mmol/ L)	2.14 + 1.81	1.40 + 0.96	3.056	0.003^*∗*^
Total cholesterol (mmol/L)	4.30 + 1.02	4.56 + 0.81	-1.726	0.086
Low-density lipoprotein cholesterol (mmol/L)	2.67 + 0.80	2.77 + 0.71	-0.849	0.397
High-density lipoprotein cholesterol (mmol/ L)	1.09 + 0.45	1.46 + 0.45	-5.113	0.080

*Note.* ^*∗*^It is used to identify the statistically significant values (*P* < 0.05).

**Table 2:** Correlation of serum 25(OH)D with renin, angiotension II, blood glucose, blood lipid, renal function, and electrolyte.

	Pearson correlation coefficient	*P* value
Renin (ng/ml)	-0.185	0.021^*∗*^
Ang II (ng/ml)	-0.062	0.442
Systolic pressure (mmHg)	-0.105	0.035^*∗*^
Diastolic pressure (mmHg)	-0.098	0.220
Blood glucose (mmol/L)	-0.004	0.958
Creatinine (umol/L)	-0.083	0.360
Triglyceride (mmol/ L)	0.051	0.529
Total cholesterol (mmol/L)	0.014	0.860
Low-density lipoprotein cholesterol (mmol/L)	0.164	0.064
High-density lipoprotein cholesterol (mmol/L)	-0.036	0.660

*Note.* ^*∗*^It is used to identify the statistically significant values (*P* < 0.05).

## Data Availability

The materials described in the manuscript, including all relevant raw data, will be made available upon request to the corresponding author.
